# An unmodified wobble uridine in tRNAs specific for Glutamine, Lysine, and Glutamic acid from *Salmonella enterica* Serovar Typhimurium results in nonviability—Due to increased missense errors?

**DOI:** 10.1371/journal.pone.0175092

**Published:** 2017-04-21

**Authors:** Kristina Nilsson, Gunilla Jäger, Glenn R. Björk

**Affiliations:** Department of Molecular Biology, Umeå University, Umeå, Sweden; Tulane University Health Sciences Center, UNITED STATES

## Abstract

In the wobble position of tRNAs specific for Gln, Lys, and Glu a universally conserved 5-methylene-2-thiouridine derivative (xm^5^s^2^U34, x denotes any of several chemical substituents and 34 denotes the wobble position) is present, which is 5-(carboxy)methylaminomethyl-2-thiouridine ((c)mnm^5^s^2^U34) in Bacteria and 5-methylcarboxymethyl-2-thiouridine (mcm^5^s^2^U34) in Eukarya. Here we show that mutants of the bacterium *Salmonella enterica* Serovar Typhimurium LT2 lacking either the s^2^- or the (c)mnm^5^-group of (c)mnm^5^s^2^U34 grow poorly especially at low temperature and do not grow at all at 15°C in both rich and glucose minimal media. A double mutant of *S*. *enterica* lacking both the s^2^- and the (c)mnm^5^-groups, and that thus has an unmodified uridine as wobble nucleoside, is nonviable at different temperatures. Overexpression of ${\text{tRNA}}_{\text{cmnm}5\text{s}2\text{UUG}}^{\text{Gln}}$ lacking either the s^2^- or the (c)mnm^5^-group and of ${\text{tRNA}}_{\text{mnm}5\text{s}2\text{UUU}}^{\text{Lys}}$ lacking the s^2^-group exaggerated the reduced growth induced by the modification deficiency, whereas overexpression of ${\text{tRNA}}_{\text{mnm}5\text{s}2\text{UUU}}^{\text{Lys}}$ lacking the mnm^5^-group did not. From these results we suggest that the primary function of cmnm^5^s^2^U34 in bacterial ${\text{tRNA}}_{\text{cmnm}5\text{s}2\text{UUG}}^{\text{Gln}}$ and mnm^5^s^2^U34 in ${\text{tRNA}}_{\text{mnm}5\text{s}2\text{UUU}}^{\text{Lys}}$ is to prevent missense errors, but the mnm^5^-group of ${\text{tRNA}}_{\text{mnm}5\text{s}2\text{UUU}}^{\text{Lys}}$ does not. However, other translational errors causing the growth defect cannot be excluded. These results are in contrast to what is found in yeast, since overexpression of the corresponding hypomodified yeast tRNAs instead counteracts the modification deficient induced phenotypes. Accordingly, it was suggested that the primary function of mcm^5^s^2^U34 in these yeast tRNAs is to improve cognate codon reading rather than prevents missense errors. Thus, although the xm^5^s^2^U34 derivatives are universally conserved, their major functional impact on bacterial and eukaryotic tRNAs may be different.

## Introduction

Transfer RNA from all organisms contains modified nucleosides, which are derivatives of the four major nucleosides adenosine (A), guanosine (G), cytidine (C), and uridine (U). More than 100 different modified nucleosides have been characterized (http://mods.rna.albany.edu or http://modomics.genesilico.pl). Although their presence in tRNA is scattered over the entire molecule, there are two positions in the tRNA that are more frequently modified than others–about 50% of nucleosides in position 34 (the wobble position) and about 80% in position 37 (next to and 3’ of the anticodon) are modified [1, 2]. Not only are these two positions frequently modified but they also contain many chemically different modifications, which are important for an efficient and accurate decoding of the mRNA (Reviewed in [1]). These modifications alter the chemical properties of the base but also the adoption of *syn* or *anti* conformations as well as inducing different tautomeric forms [3]. Unmodified U is present rarely in this position of tRNAs from free living organisms [2]. Apparently, a modified U in the wobble position of tRNAs of most cellular organisms is required for efficient decoding with required fidelity.

**Fig 1:**
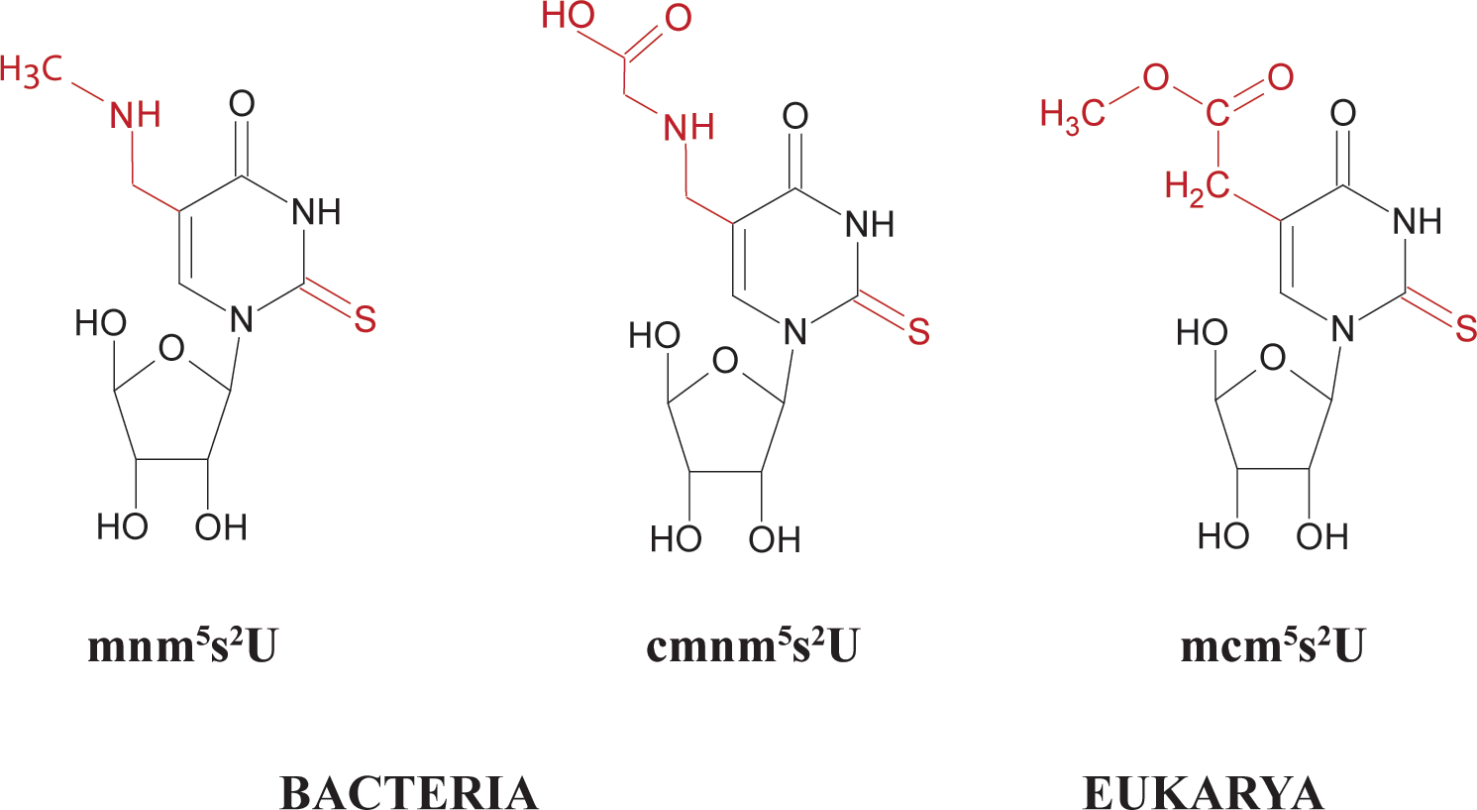
Structures of the nucleosides present in the wobble position of tRNAs specific for Gln, Lys, and Glu in Bacteria [mnm^5^s^2^U (Lys and Glu) and cmnm^5^s^2^U (Gln)] and in Eukarya (mcm^5^s^2^U).

**Fig 2:**
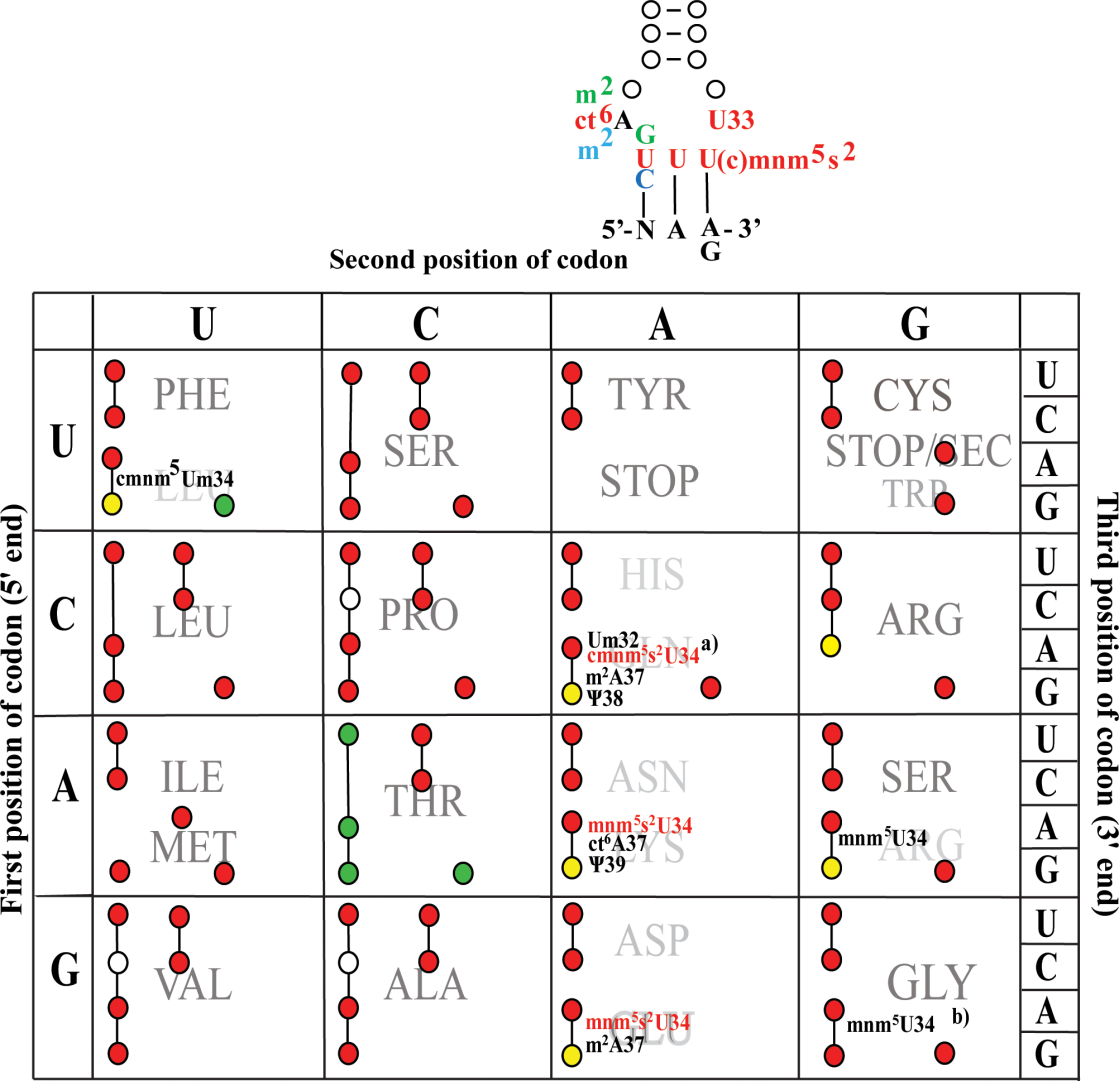
Codon table and the anticodon loop of tRNAs specific for Gln, Lys, and Glu which contain cmnm^5^s^2^U (Gln) or mnm^5^s^2^U (Lys and Glu) as wobble nucleoside in bacterial tRNAs. Above the third column (second codon base A) the anticodon stem and loop of the tRNAs containing the (c)mnm^5^s^2^U in position 34 is shown (denoted in red **cmnm5s2**). Position 36 of the anticodon is color coded with Green **G36** being position 36 for ${\text{tRNA}}_{\text{cmnm}5\text{s}2\text{UUG}}^{\text{Gln}}$, red **U36** for ${\text{tRNA}}_{\text{mnm}5\text{s}2\text{UUU}}^{\text{Lys}}$, and blue **C36** for ${\text{tRNA}}_{\text{mnm}5\text{s}2\text{UUU}}^{\text{Glu}}$. Similar color code is denoted for the modified nucleosides present in position 37 in the corresponding tRNAs. N denotes G, A or C, respectively, for first nucleoside in the relevant codons read by these tRNAs. Note that these tRNAs are rich in U, which is a poor stacker [24] making the anticodon very flexible especially if the modifications is absent and this is especially true of ${\text{tRNA}}_{\text{mnm}5\text{s}2\text{UUU}}^{\text{Lys}}$. In the codon table the letters outside the box, to the left, above, and to the right indicate the first, second, and third position of the codon. Circles connected by a line, or a single circle, represent one tRNA species. A filled circle indicates the capacity of that tRNA to base pair with the indicated codon, either by Watson-Crick or by wobble according to the revised wobble hypothesis [1]. Red and yellow circles indicate tRNAs that are sequenced at the RNA level while green circles represent tRNAs for which only a partial tRNA sequence is available. A red or green circle indicates efficient base pairing while a yellow circle indicates a restricted wobble. An open (white) circle is a base pairing that is not according to the revised wobble hypothesis. However, data *in vivo* from mutants where only this tRNA is left to decode all codons in the codon box, suggest that the tRNA in fact is able to read that codon [9, 25]. Data are compiled from Sprinzl data base (http://trnadb.bioinf.uni-leipzig.de/ and Modomics data base (http://modomics.genesilico.pl/). Mutations in *mnmE* or *mnmG* genes result in no formation of the (c)mnm^5^- group not only of the (c)mnm^5^s^2^U present in tRNAs specific for Gln, Lys and Glu but also cmnm^5^Um in ${\text{tRNA}}_{\text{cmnm}5\text{UAA}}^{\text{Leu}}$ and mnm^5^U34 in tRNAs specific for Arg and Gly. Mutations in *mnmA* results in no thiolation of cmnm^5^s^2^U34. **a**) The modifications in position 34 of ${\text{tRNA}}_{\text{cmnm}5\text{s}2\text{UUG}}^{\text{Gln}}$ from *S*. *enterica* are cmnm^5^s^2^U (80%) and mnm^5^s^2^U (20%) [10] and similar in *E*. *coli* [11] **b**) The majority of this tRNA^Gly^ contains mnm^5^U34 but there is also a small amount of cmnm^5^U34 [1].

Modified wobble U’s are classified in two groups based on their chemical structures. One group consists of 5-hydroxyuridine derivatives with a chemical group attached at position 5 of the uracil base via an ether linkage (xo^5^U34-derivatives) whereas the other group consists of 5-mehyluridine derivatives (xm^5^U34-derivatives) with a methylene carbon attached to the C5 atom of uracil (x denotes any of several different chemical substituents). The xo^5^U34- derivatives are found in tRNAs reading family codon boxes and the modification expands wobble capacity of uridine to read 3 to 4 codons in such a codon box. The xm^5^U34-derivatives are present in tRNAs reading mixed codon boxes and decode codons ending with A or G. They may also have a sulfur bound to position 2 of uracil (xm^5^s^2^U34-derivatives; reviewed in [1]). These wobble derivatives (xm^5^s^2^U34) are universally conserved in tRNAs specific for Gln, Lys, and Glu and, accordingly, are present in the three phylogenetic domains Bacteria, Eukarya and Archaea and also in organelles such as mitochondria and chloroplasts. The 5-(carboxy)methylaminomethyl-2-thiouridine ((c)mnm^5^s^2^U34) derivative of these modifications are present as wobble nucleosides in bacterial tRNAs specific for glutamine (Gln), lysine (Lys), and glutamic acid (Glu) whereas the wobble nucleoside in the eukaryotic tRNA counterparts is 5-methylcarboxymethyl-2-thiouridine (mcm^5^s^2^U34) (Fig 1). Such derivatives were thought to restrict the wobble capacity of U and thereby improving the recognition of purine-ending codons and prevent misreading of the near-cognate codons ending in pyrimidines [4] (cf. Fig 2). The synthesis of the latter in yeast requires the activity of several genes, of which ELP3 is required for the early step(s) in the synthesis of the mcm^5^-side chain and TUC1 is required for last step in the thiolation at position 2 [5, 6]. The yeast double mutant *elp3*, *tuc1*, which thus contains an unmodified wobble uridine in tRNAs specific for Gln, Lys, and Glu, is nonviable but can be rescued by overexpression of the corresponding hypomodified tRNAs. The latter result suggests that the primary function of mcm^5^s^2^U34 is to improve the efficiency of decoding cognate codons rather than to prevent missense errors [5].

In bacteria the functional aspect of (c)mnm^5^s^2^U34 is more complex. Although it was thought that the function of these modifications was to prevent misreading, Hagervall et al [7] showed that this is not the case for ${\text{tRNA}}_{\text{mnm}5\text{s}2\text{UUU}}^{\text{Lys}}$, since lack of either the mnm^5^- or the s^2^-group reduces misreading of the near-cognate Asn-codons (AAU/C, see Fig 2). Moreover, Manickam et al [8] confirm these results for the lack of the mnm^5^- side chain and further show that such hypomodified ${\text{tRNA}}_{\text{mnm}5\text{s}2\text{UUU}}^{\text{Lys}}$ also decreases other missense errors (Arg codons AGA/G; see Fig 2). However, lack of mnm^5^-side chain in ${\text{tRNA}}_{\text{mnm}5\text{s}2\text{UUU}}^{\text{Glu}}$ increases missense errors of Gly (GGA) and Asp (GAU/C) codons [8]. Thus, the same modification (mnm^5^-side chain) in ${\text{tRNA}}_{\text{mnm}5\text{s}2\text{UUU}}^{\text{Lys}}$ or in ${\text{tRNA}}_{\text{mnm}5\text{s}2\text{UUU}}^{\text{Glu}}$ influences the frequency of missense errors differently–increasing (${\text{tRNA}}_{\text{mnm}5\text{s}2\text{UUU}}^{\text{Lys}}$) or decreasing (${\text{tRNA}}_{\text{mnm}5\text{s}2\text{UUU}}^{\text{Glu}}$). Therefore, the functional impact of the modification is sensitive to the structural context it is part of, which has also been shown for the modified nucleosides queosine (Q34) [8] and the uridine-5-oxyacetic acid (cmo^5^U34) [9].

**Fig 3:**
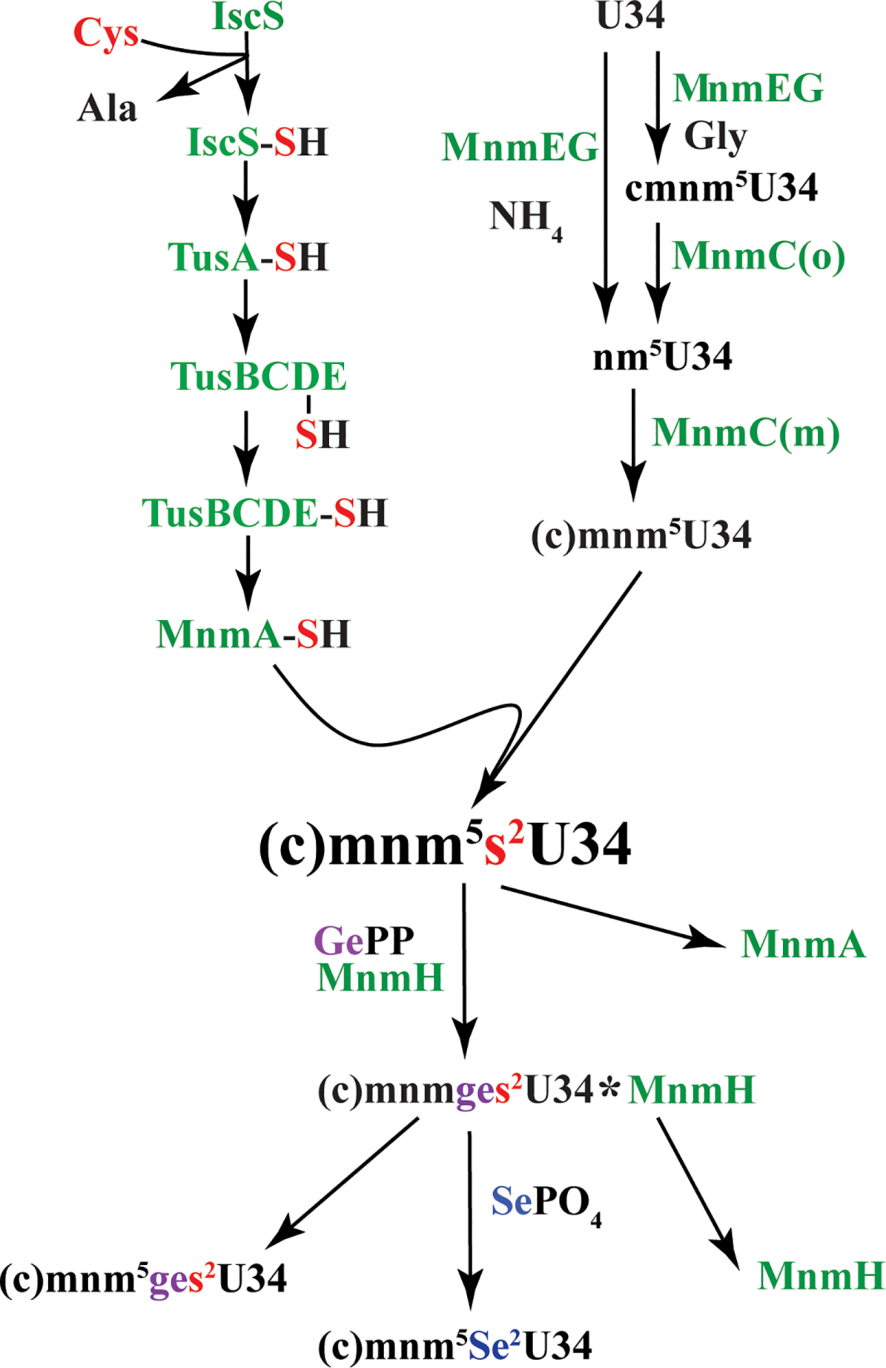
Synthesis of (c)mnm^5^s^2^U in bacteria. Note that a mutations in *mnmA*, *tusB* or *tusE* block the formation of the s^2^-group and mutations in either *mnmE* or *mnmG* (*gidA*) block the synthesis of the side chain (c)mnm^5^-. In the bacterium *Salmonella enterica* Serovar Typhimurium LT2, the sulfur may be exchanged by selenium, depending on the concentration of selenium in the growth medium. The intermediate in the selenation process is a geranylated derivative (c)mnm^5^ges^2^U34 (ge, denotes a geranyl group covalently bound to the s^2^-group) and it is present in the wild type bacteria at a level of only a few percent [26].

Transfer RNA specific of Gln, Lys, and Glu are the only tRNA species in bacteria that contain (c)mnm^5^s^2^U34 as wobble nucleoside. Whereas ${\text{tRNA}}_{\text{cmnm}5\text{s}2\text{UUG}}^{\text{Gln}}$ contains a mixture of cmnm^5^s^2^U (80%) and mnm^5^s^2^U (20%) [10–12], ${\text{tRNA}}_{\text{mnm}5\text{s}2\text{UUU}}^{\text{Lys}}$ and ${\text{tRNA}}_{\text{mnm}5\text{s}2\text{UUU}}^{\text{Glu}}$ contain mnm^5^s^2^U34. The formation of (c)mnm^5^s^2^U34 requires the activity of several enzymes as summarized in Fig 3. A predicted minimal translation apparatus requires the *mnmA*, *mnmE* and *mnmG* genes but not the *mnmC* gene to synthesize cmnm^5^s^2^U34, which suggests an important role of this wobble modification for a proper translation [13]. The s^2^- and the (c)mnm^5^-group of (c)mnm^5^s^2^U34 are pivotal for reading frame maintenance and lack of either of them induces ability to suppress frameshift mutations [14]. We have characterized several frameshift suppressor mutants lacking this modified nucleoside and these mutants are our tools in studying the function of the (c)mnm^5^s^2^U34 wobble nucleoside [15]. Mutations in *mnmA*, *tusB*, or *tusE* block the sulfur relay pathway whereas mutations in *mnmE* or *mnmG* (*gidA*) block the synthesis of the (c)mnm^5^-side chain and accordingly such mutants lack (c)mnm^5^s^2^U34 (Fig 3, Table 1).

**Table 1:** Level of (c)mnm^5^s^2^U in tRNAs of various strains used in this study.

Strain	Relevant genotype	s^2^C/Ψ 254 nm	mnm^5^s^2^U/Ψ ^a^ 254 nm	s^2^U/ Ψ 254 nm	(c)mnm^5^s^2^U/s^4^U 314 nm	s^2^U/s^4^U 314 nm
GT7321	*mnmA*^*+*^, *mnmE*^*+*^	0.10	0.042	<0.001	0.047	<0.001
GT8173	*mnmA16<>cat*	0.12	<0.001	<0.001	<0.003	<0.002
GT8176	*mnmE17<>kan*	0.12	<0.003	0.048 ^b^	<0.002	0.019
GT8177	p*mnmA*^*+*^*/ mnmA16<>cat* ^c^	0.12	0.054	<0.001	0.046	<0.002
GT7440	*mnmA3* (G24D)	0.12	< 0.001	d	< 0.001	D
GT7453	*tusB27* (Q31stop)	0.12	< 0.001	d	< 0.001	D
GT7432	*tusE30* (K128stop)	0.11	< 0.003	d	< 0.002	D
GT7436	*mnmE13* (codon 241–271 deleted)	0.14	< 0.001	d	< 0.001	D
GT7478	*mnmG1*(*gidA1*, 58 nt deletion from A402)	0.13	<0.002	d	< 0.001	D

**Fig 4:**
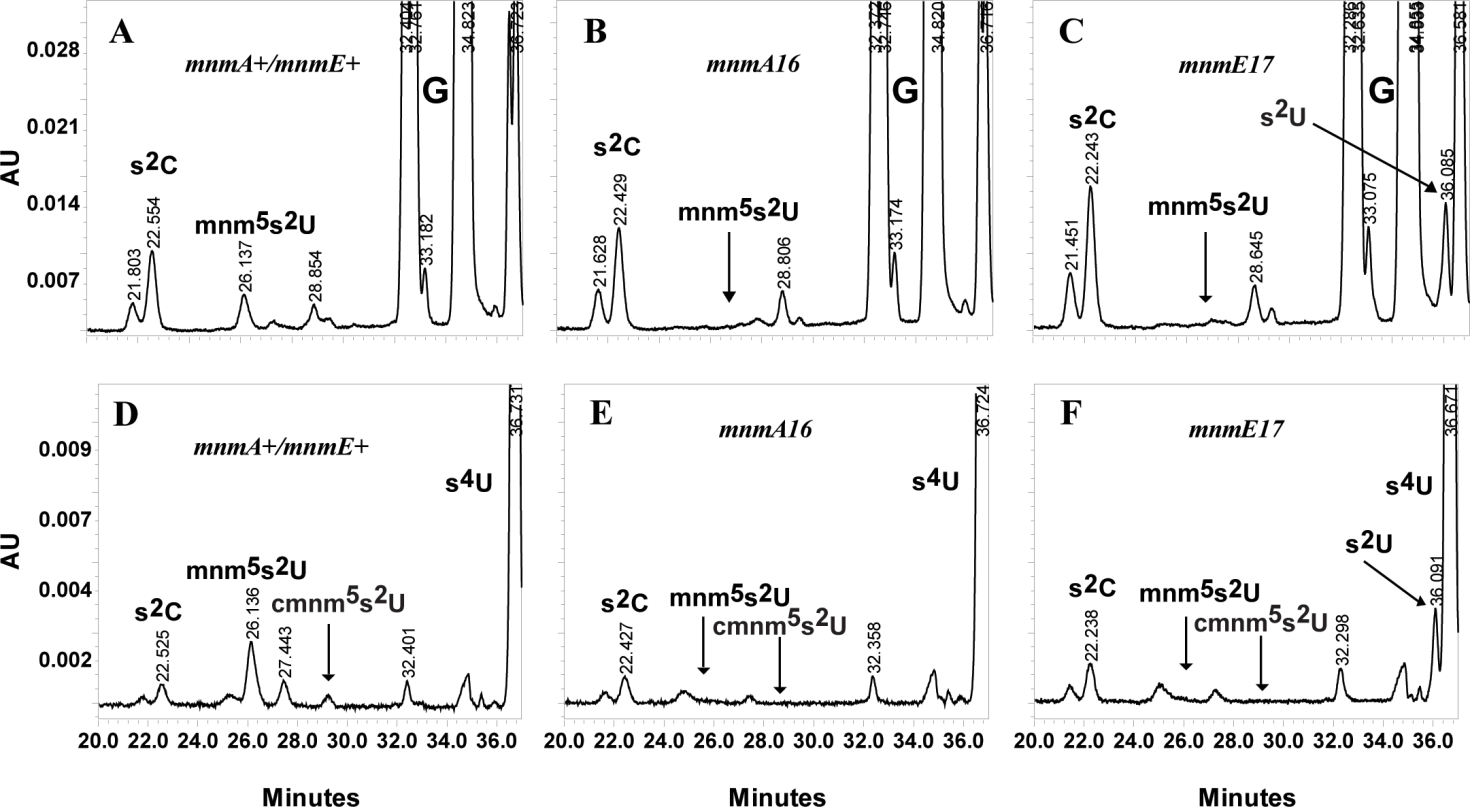
HPLC analysis of tRNA from *mnmE17<>Km* and *mnmA16<>cat* mutants grown in rich medium at 37°C. (**A**) Strain GT7132 (*mnmA*^*+*^, *mnmE*^*+*^). (**B**) Strain GT8173 (*mnmA16*<>cat). (**C**) Strain GT8176 (*mnmE17*<>kan). (**D)** Strain GT7132 (*mnmA*^*+*^, *mnmE*^*+*^). (**E**) Strain GT8173(*mnmA16*<>cat). (**F**) Strain GT8176 (*mnmE17*<>kan). Panel A,B and C are monitored at 254 nm and panel D,E and F are monitored at 314 nm. *AU*, absorbance units.

a) The cmnm5s2U peak is hidden under contaminating material, so it is not possible to determine the area.

b) This level is likely to be an overestimate due to poor separation from especially G. (See Fig 4C). The level is about 100% of the average level of mnm^5^s^2^U in the two wild type strains GT7321 and GT8177.

c) Strain GT8177 contains a plasmid from the Saka collection,; [45], which harbors the wild type allele of *mnmA*^*+*^ gene and the *mnmA16<>cat* insertion on the chromosome.

d) These analysis were made using a Supelco C-18 column, which does not separate s^2^U from s^4^U. However, this analysis determines excellently the level of mnm^5^s^2^U at 254 nm and both cmnm^5^s^2^U and mnm^5^s^2^U at 314 nm.

This paper focus on the functional aspect of the wobble nucleoside (c)mnm^5^s^2^U34 in bacteria. We show here that lack of either the s^2^- or the (c)mnm^5^-group induces poor growth and cold sensitivity, that a double mutant having an unmodified U34 as wobble nucleoside is not viable, and that overexpression of ${\text{tRNA}}_{\text{cmnm}5\text{s}2\text{UUG}}^{\text{Gln}}$ or ${\text{tRNA}}_{\text{mnm}5\text{s}2\text{UUU}}^{\text{Lys}}$ lacking the s^2^-group reduces growth considerable suggesting that such hypomodified tRNAs increase missense error. Overexpression of ${\text{tRNA}}_{\text{cmnm}5\text{s}2\text{UUG}}^{\text{Gln}}$ lacking the cmnm^5^-group reduces growth although much less than s^2^-deficiency, whereas lack of the mnm^5^ group of overexpressed ${\text{tRNA}}_{\text{mnm}5\text{s}2\text{UUU}}^{\text{Lys}}$ does not.

## Materials and methods

### Bacteria and growth conditions

The bacterial strains used were derivatives of *Salmonella enterica* serovar Typhimurium LT2 or *Escherichia coli* K12 (S1 Table, Supporting materials). As rich medium Luria-Bertani (LB) was used [16]C:\GetARef\Refs\Refsmanus\FS_mutant_2011.ref #2; C:\GetARef\Refs\Refsmanus\Kristina-Gln05.ref #38;.The minimal solid medium was made from Vogel & Bonner basal salt medium [17] C:\GetARef\Refs\Refsmanus\FS_mutant_2011.ref #4; C:\GetARef\Refs\Refsmanus\Kristina-Gln05.ref #39; with 15g of agar per liter and supplemented with 0.2% glucose and required amino acids and/or vitamins [18]C:\GetARef\Refs\Refsmanus\FS_mutant_2011.ref #5; C:\GetARef\Refs\Refsmanus\Kristina-Gln05.ref #40;. When necessary antibiotics were added at following concentrations: carbenicillin 100 μg ml^-1^, kanamycin 50 μg ml^-1^ and chloramphenicol 25 μg ml^-1^.

### Genetic procedures

Transduction with phage P22 HT105/1 (*int-201*) [19]C:\GetARef\Refs\Refsmanus\FS_mutant_2011.ref #20; was performed as previously described [18]C:\GetARef\Refs\Refsmanus\Kristina-Gln05.ref #40;. DNA sequencing was performed on plasmid DNA or PCR products following the manual of Applied Biosystems ABI PRISM Cycle Sequencing Ready Reaction Kit Big Dye^TM^ v.1.1 or by LightRun^TM^ sequencing at GATC Biotech, Cologna, Germany. The inactivation of *mnmA* and *mnmE* genes was done according to Datsenko & Wanner [20] and the resulting insertions were confirmed by PCR.

### Analysis of modified nucleosides in tRNA

Bacterial strains were grown over night in medium LB, diluted 100 times in 100 ml of the same medium and grown at 37°C to a cell density of about 4x10^8^ cells/ml. Cells were lysed and total RNA was prepared [21] and dissolved in 2 ml buffer R200 (10 mM Tris-H_3_PO_4_ (pH 6.3), 15% ethanol, 200 mM KCl) and applied to a Nucleobond^®^ AX500 column (Macherey-Nagel Gmbh & Co., Düren, Germany), pre-equilibrated with the same buffer. The column was washed once with 6 ml R200 and once with 2 ml R650 (same composition as R200, except for 650 mM KCl instead of 200 mM KCl). Finally, tRNA was eluted with 7 ml R650, precipitated by 0.7 volumes cold isopropanol, washed twice with 70% ethanol and dissolved in water. Transfer RNA was digested to nucleosides by nuclease P1 followed by treatment with bacterial alkaline phosphatase at pH 8.3 [22]. C:\GetARef\Refs\Jocke\cmo5U.ref #91; The hydrolysate was analyzed as described earlier [23] using a Supelcosil C-18 column (Supelco) or Develosil C-30 (Phenomenex) with a Waters Alliance HPLC system.

## Results

### The *mnmA* or *mnmE* mutants have undetectable level of (c)mnm^5^s^2^U34 in tRNA

To test the viability of a mutant containing mutations in both *mnmA* and *mnmE*, selectable markers linked to the inactivation of these genes were required. We therefore inserted in the *mnmA* gene a chloramphenicol (*cat*) resistant element and in the *mnmE* gene a kanamycin (*Km*) resistant element (denoted *mnmA16<>cat* and *mnmE17<>Km*, respectively), which completely destroy the synthesis of the corresponding enzymes and block the synthesis of (c)mnm^5^s^2^U34. In an *mnmA16<>cat*, *mnmE17<>Km* double mutant tRNAs specific for Gln, Lys and Glu will therefore contain an unmodified wobble uridine (U34). These resistance elements should not induce any polar effect on the expression on downstream genes, since both genes are transcribed as single cistrons (http://regulondb.ccg.unam.mx/).

The biosynthesis of the cmnm^5^- and the s^2^-groups are suggested to be independent of each other [27–29]. Accordingly, a deletion of the *mnmE* or the *mnmG* gene should result in tRNAs specific for Gln, Lys and Glu containing s^2^U34 instead of (c)mnm^5^s^2^U (Fig 3). If the thiolation is not dependent of the presence of the (c)mnm^5^ side chain, a similar level of moles of s^2^U should accumulate as the level of (c)mnm^5^s^2^U34 present in the wild type. Indeed, s^2^U is observed in the *mnmE17<>Km* mutant but at a level of 40% (at 314 nm) and 100% (at 254 nm) of the level of (c)mnm^5^s^2^U in the wild type (assuming the same extension coefficient constants for (c)mnm^5^s^2^U and s^2^U; Table 1, Fig 4). Note, that s^2^U is not well separated from s^4^U at 314 nm and G at 254 nm (Fig 4C and 4E) making the analysis not optimal. However, analysis of tRNA of an *mnmE* mutant of *E*. *coli* s^2^U is also about 50% of the level of mnm^5^s^2^U in the wild type at analysis conditions where s^2^U is well separated from s^4^U [30]. On the other hand determinations using radioactive labelling have found higher level of s^2^U in relation to the level of (c)mnm^5^s^2^U (80% by Hagervall et al [11]). Although the *mnmE* mutant used has s^2^U in its tRNA we cannot rule out that the level might not reach the expected level even though thiolation, as has been suggested, is not dependent on the presence of the cmnm^5^-side chain.

A mutation in the *mnmA*, *tusB* or *tusE* genes should result in the presence of cmnm^5^U34 in the Gln- and mnm^5^U34 in Lys- and Glu-tRNAs (cf Fig 3). However, in the HPLC analysis used the mnm^5^U is not separated from the major nucleoside C. However, we have earlier analysed the distribution of modified nucleosides in an *mnmA* mutant and such a mutant contains mnm^5^U34 instead of mnm^5^s^2^U34 [7]. Moreover, *mnmA*, *tusB* or *tusE* deletion mutants contain mnm^5^U34 in tRNA instead of mnm^5^s^2^U34 [31]. Since the (c)mnm^5^s^2^U is not detected in tRNA of the *mnmA*, *tusB* and *tusE* mutants used (Table 1), it is likely that they instead have (c)mnm^5^U in their tRNAs specific to Gln-, Lys-, and Glu.

### Lack of the s^2^- or (c)mnm^5^-group of (c)mnm^5^s^2^U as in the *mnmA* and *mnmE* mutants, respectively, results in a severe growth reduction especially at low temperature

As described in the introduction yeast has the chemically related wobble nucleoside mcm^5^s^2^U in the corresponding tRNA species in which (c)mnm^5^s^2^U34 is present in *S*. *enterica* and *E*. *coli* (Fig 1). The Elp3p catalyses the first step in the synthesis of the mcm^5^-group and the Tuc1p the last step in synthesis of the s^2^-group [5, 6]. A double mutant *elp3*, *tuc1* is nonviable demonstrating that an unmodified U34 in the Gln, Lys and Glu-tRNA does not support viability in yeast [5]. However, lowering the temperature allows such a double mutant to grow, although poorly. Assuming that a bacterial *mnmA*,*mnmE* double mutant is also viable at lower temperature, we monitored the growth of the *mnmA3* and *mnmE13* single mutants at several temperatures and growth media. However, both single mutants grew very poorly at all conditions tested, especially at low temperature, and not at all at 15°C [on rich (*mnmA* and *E* mutants) and minimal medium (*mnmA* mutant)] (Table 2). The reduction of growth at 37°C is similar to the growth behaviour of *mnmA*, *tusB* and *tusE* mutants described by Suzuki and collaborators [31].

**Table 2:** The *mnmA* and mnmE mutants are cold sensitive on rich and minimal glucose media.

LAL (relative colony size)				Glucose+His (relative colony size)			
Temp	GT7321 (wt)	GT8176 (*mnmE17<>Km*)	GT8173 (*mnmA16 <>cat*)	Temp	GT7321 (wt)	GT8176 (*mnmE17<>Km*)	GT8173 (*mnmA16<>cat*)
42.5^a)^	**1.0 (**1.7)	0.7	<0.1	41^b)^	**1.0** (1.8)	0.39	0.33
37^a)^	**1.0** (1.2)	0.75	0.5	37^b)^	**1.0** (2.0)	0.7	0.55
30^b)^	**1.0** (3.2)	0.16	no sc	30^b)^	**1.0** (1.5)	0.67	< 0.1
RT^b)^	**1.0** (1.1)	0.45	no sc	RT^c)^	**1.0** (0.5)	<0.1	no sc < 0.1 (6d)
15^d)^	**1.0** (1.0)	< 0.1	no sc < 0.1 (14 d)	15^e)^	**1.0** (0.9)	1.0–1.8 (13 d)	no sc (13 d)

Values are given as colony size (in mm; average of 10 isolated colonies) relative to that of the size of the wild type following the same time of incubation. The colony size of the wild type is given within parenthesis. <0.1, colonies less than 0.1 mm; no sc: no single cell colonies was observed and growth was observed only on primary and secondary streaks. The size of the colony was determined under a magnifying glass and its diameter was determined using a slide calliper. (LAL-plates, rich media, Glucose+His–plates with minimal media)

a), scored after one day

b) scored after two days

c) scored after three days

d) scored after four days and

e) scored after six days. When the time of incubation of the plates was longer than that for the wild type, the number of days are indicated within parenthesis.

### Overexpression of Gln or Lys specific tRNAs lacking the 2-thio- or the (c)mnm^5^- group of (c)mnm^5^s^2^U34 causes severe growth reduction

In a yeast *elp3*, *tuc1* double mutant tRNAs specific for Gln, Lys, and Glu lack mcm^5^s^2^U34 in their wobble positions and have instead an unmodified U34. Such a double mutant is not viable, but overexpression of hypomodified versions of these tRNAs rescues the double mutant *elp3*, *tuc1* [5]. A corresponding bacterial *mnmA*, *mnmE* double mutant, which should also have an unmodified wobble U34 in the corresponding tRNAs, would therefore be potentially nonviable. Since the xm^5^s^2^U34 derivatives are universally conserved their function might be similar so that overexpression of bacterial hypomodified tRNAs might, as in yeast, rescue a potential nonviable double mutant *mnmA*, *mnmE*. Overexpression of a hypomodified tRNA (e.g hypomodified ${\text{tRNA}}_{\text{cmnm}5\text{s}2\text{UUG}}^{\text{Gln}}$) in the recipient cell might therefore facilitate the construction of a viable mutant having both *mnmA* and *mnmE* mutations on the chromosome and a rescuing plasmid encoding e.g. hypomodified ${\text{tRNA}}_{\text{cmnm}5\text{s}2\text{UUG}}^{\text{Gln}}$ or ${\text{tRNA}}_{\text{mnm}5\text{s}2\text{UUU}}^{\text{Lys}}$. We therefore first monitored how overexpression of a hypomodified ${\text{tRNA}}_{\text{cmnm}5\text{s}2\text{UUG}}^{\text{Gln}}$ or ${\text{tRNA}}_{\text{mnm}5\text{s}2\text{UUU}}^{\text{Lys}}$ influenced growth of the *mnmA3* and *mnmE13* mutants.

#### Overexpression of ${\mathrm{tRNA}}_{\mathrm{cmnm}5s2\mathrm{UUG}}^{\mathrm{Gln}}$ lacking the s^2^-group and possessing only the cmnm^5^-group of cmnm^5^s^2^U34

**Fig 5:**
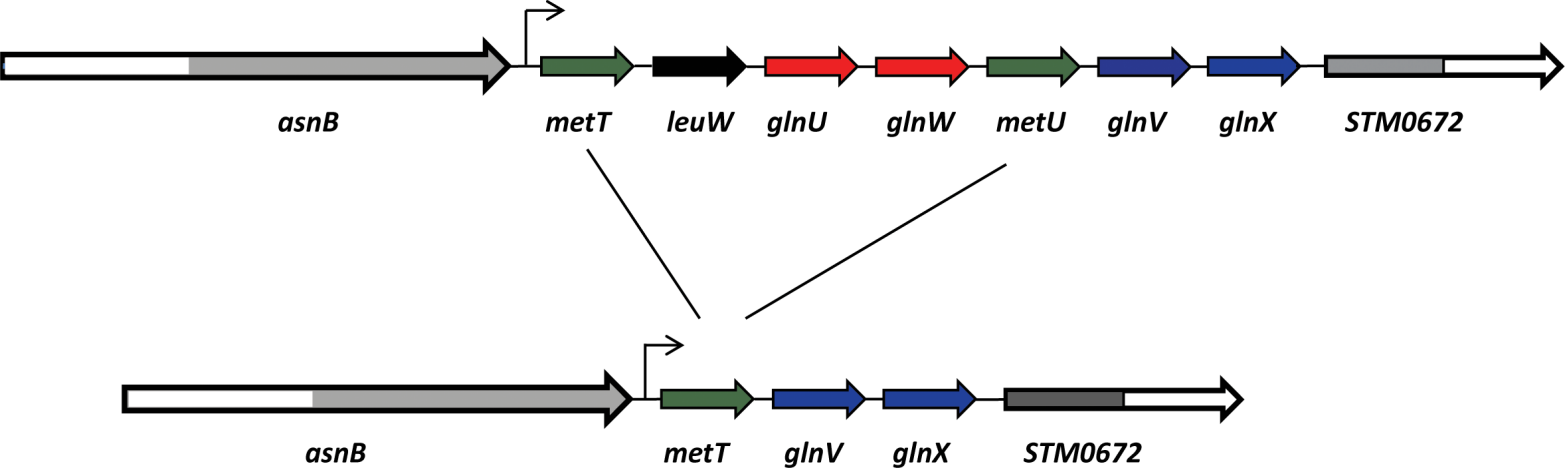
Gene organization of plasmids pUST313 and pUST314. Plasmid pUST313 harbors the genes *glnU* and *glnW* (**red**) encoding the (c)mnm^5^s^2^U34 containing Gln-tRNA (${\text{tRNA}}_{\text{cmnm}5\text{s}2\text{UUG}}^{\text{Gln}}$) and genes *glnV* and *glnX* (**blue**) encoding the C34 containing ${\text{tRNA}}_{\text{CUG}}^{\text{Gln}}$. Plasmid pUST314 is a spontaneous plasmid mutant in which a recombination has occurred between *metT* (${\text{tRNA}}_{\text{ac}4\text{CAU}}^{\text{Met}}$) and *metU* (also ${\text{tRNA}}_{\text{ac}4\text{CAU}}^{\text{Met}}$) (**green**) resulting in a deletion of the *glnU* and *glnW* genes encoding ${\text{tRNA}}_{\text{cmnm}5\text{s}2\text{UUG}}^{\text{Gln}}$ and *leuW* (**black**). Note, only part of the *asnB* and STMO672 genes are present (shaded gray) suggesting that these defective genes do not express any full sized protein. Plasmid p815 (not shown in the figure but used in some experiments) contains the *valU* operon, which consists of genes *valU*, *valX*, *valY* and *lysV*. The latter is the structural gene for ${\text{tRNA}}_{\text{mnm}5\text{s}2\text{UUU}}^{\text{Lys}}$.

Strains having a mutation in *mnmA*, *tusB* or *tusE* genes lack the s^2^-group of (c)mnm^5^s^2^U34 of ${\text{tRNA}}_{\text{cmnm}5\text{s}2\text{UUG}}^{\text{Gln}}$ and such a tRNA has only the cmnm^5^-group (See above). Plasmid pUST313 harbors the *metT* operon consisting of genes *glnU* and *glnW* encoding the (c)mnm^5^s^2^U34 containing ${\text{tRNA}}_{\text{cmnm}5\text{s}2\text{UUG}}^{\text{Gln}}$ and genes *glnV* and *glnX* encoding the C34 containing ${\text{tRNA}}_{\text{CUG}}^{\text{Gln}}$. This operon also contains the *metT/U* genes encoding ${\text{tRNA}}_{\text{ac}4\text{CAU}}^{\text{Met}}$ and the *leuW* gene encoding ${\text{tRNA}}_{\text{cmo}5\text{UAG}}^{\text{Leu}}$ (Fig 5). Note, no other full sized gene is present on this plasmid. We introduced this plasmid into *mnmA3*, *tusB27 and tusE30* mutants as well as into the wild type strain. Overexpression of ${\text{tRNA}}_{\text{cmnm}5\text{s}2\text{UUG}}^{\text{Gln}}$ from plasmid pUST313 in the wild type influenced the growth only to a minor degree (Table 3). Slow growth as such was not sensitive to the overexpression of fully modified ${\text{tRNA}}_{\text{cmnm}5\text{s}2\text{UUG}}^{\text{Gln}}$ as tested by introducing the same plasmid into a mutant having a similar growth rate as an *mnmA* mutant (data not shown). However, plasmid pUST313 severely reduced the growth of the three independent mutants *mnmA3*, *tusB27* and *tusE30* all lacking the s^2^- group of (c)mnm^5^s^2^U34 in ${\text{tRNA}}_{\text{cmnm}5\text{s}2\text{UUG}}^{\text{Gln}}$. Since the results were the same in the three independent s^2^-deficieint mutants, it was the lack of the s^2^-group of (c)mnm^5^s^2^U in ${\text{tRNA}}_{\text{cmnm}5\text{s}2\text{UUG}}^{\text{Gln}}$ that caused the reduced growth and no other aberrations in the cell. We obtained a few large colonies among the small colonies when introducing plasmid pUST313 into the *mnmA3* mutant. Sequencing the *metT* operon of the plasmid present in such a large spontaneously occurring colony revealed that a recombination had occurred between the plasmid encoded *metT* and *metU* genes resulting in the loss of genes *glnU* and *glnW*, which code for ${\text{tRNA}}_{\text{cmnm}5\text{s}2\text{UUG}}^{\text{Gln}}$ (Fig 5). The resulting plasmid, pUST314, therefore lacks these genes and have only *metT/U* gene encoding ${\text{tRNA}}_{\text{ac}4\text{CAU}}^{\text{Met}}$ and *glnV* and *glnX* genes encoding the C34 containing ${\text{tRNA}}_{\text{CUG}}^{\text{Gln}}$. This plasmid caused only a 50% growth reduction of the *mnmA3* and the *tusB27* mutants compared to the almost complete inhibition of growth caused by the ${\text{tRNA}}_{\text{cmnm}5\text{s}2\text{UUG}}^{\text{Gln}}$ expressing plasmid pUST313. Thus, a substantial part of the growth reduction is caused by overexpression of ${\text{tRNA}}_{\text{cmnm}5\text{s}2\text{UUG}}^{\text{Gln}}$ (Table 3). Plasmid pUST314 also reduced growth suggesting that overexpression of ${\text{tRNA}}_{\text{ac}4\text{CAU}}^{\text{Met}}$ and of ${\text{tRNA}}_{\text{CUG}}^{\text{Gln}}$ had some growth reducing effect in strains lacking the s^2^-group of (c)mnm^5^s^2^U34 but not at all in the wild type strain. In summary, overexpression of s^2^U-deficient ${\text{tRNA}}_{\text{cmnm}5\text{s}2\text{UUG}}^{\text{Gln}}$ in three independent mutants (*mnmA3*, *tusB27* and *tusE30*) induced a severe growth reduction that was not observed if this tRNA was not overexpressed.

**Table 3:** Overexpression of hypomodified ${\mathrm{tRNA}}_{\mathrm{cmnm}5s2\mathrm{UUG}}^{\mathrm{Gln}}$ or ${\mathrm{tRNA}}_{\mathrm{mnm}5s2\mathrm{UUU}}^{\mathrm{Lys}}$ severely reduced cellular growth.

Relevant genotype on chromosome	Wobble nucleoside in tRNA Gln, Lys, and Glu	Plasmid (Relevant genotype)	Growth on LAL- plates. (Rel. colony size (1 d))	Growth on His-glucose–plates. (Rel. colony size (2 d)).
Wild type	(c)mnm^5^s^2^U34	No plasmid	0.9	1.0;
		pUST312 (vector)	**1.0; 1.0**	**1.0 1.0**
		pUST313 (*glnU*,*X*^a)^)	0.8; 0.8	0.6; 0.8
		pUST314 (*ΔglnU*,*X*^b)^)	1.0;	0.9;
		p815 (*lysV*^c)^)	0.9	1.0
*mnmA3*	(c)mnm^5^U34(lacks the s^2^-group)	No plasmid	1.1	1.1
		pUST312 (vector)	**1.0; 1.0**	**1.0, 1.0**
		pUST313 (*glnU*,*X*^a)^)	No sc; <0.1	<0.1; <0.1
		pUST314 (*ΔglnU*,*X*^b)^)	0.6;	<0.1
		p815 (*lysV*^c)^)	0.2	0.1
*tusB27*	(c)mnm^5^U34(lacks the s^2^-group)	No plasmid	0.9	1,0
		pUST312 (vector)	**1.0; 1.0**	**1.0**
		pUST313 (*glnU*,*X*^a)^)	No sc; No sc	<0.1; No sc
		pUST314 (*ΔglnU*,*X*^b)^)	0.5;	0.1;
		p815 (*lysV*^c)^)	0.2	0.1
*tusE30*	(c)mnm^5^U34(lacks the s^2^-group)	No plasmid	0.9	1.1
		pUST312 (vector)	**1.0**	**1.0**
		pUST313 (*glnU*,*X*^a)^)	No sc; No sc	0.2; No sc
		pUST314 (*ΔglnU*,*X*^b)^)	ND	ND
		p815 (*lysV*^c)^)	0.2	<0.1
*mnmE13*	s^2^U34(lacks the (c)mnm^5^-group	No plasmid	0.9	1.0
		pUST312 (vector)	**1.0; 1.0**	**1.0; 1.0**
		pUST313 (*glnU*,*X*^a)^)	0.4; 0.4	<0.1; No sc
		pUST314 (*ΔglnU*,*X*^b)^)	0.9;	0.8
		p815 (*lysV*^c)^)	1.2	1.0
*mnmG1*	s^2^U34(lacks the (c)mnm^5^-group)	No plasmid	0.9	1.1
		pUST312 (vector)	**1.0; 1.0**	**1.0: 1.0**
		pUST313 (*glnU*,*X*^a)^)	0.5; 0.4	0.4; 0.2
		pUST314 (*ΔglnU*,*X*^b)^)	ND	ND
		p815 (*lysV*^c)^)	1.2	1.0

Indicated strains were grown at 37°C in the rich media LB overnight and then streaked out on LAL agar plates or on glucose medium containing histidine, since the host strain GT7321 requires histidine for growth. No sc, denotes that no single colonies were observed and growth only on primary and/or secondary outstreaks were observed. Numbers are the average of 10 similar sized colonies after indicated time of incubation. When two numbers are present the experiment was repeated once. The size of the colonies was determined a described in Table 2. ND = not done

a) Plasmid pUST313 carry the *metT* operon which contains the tRNA genes: *metT-leuW-glnU-glnW-metU-glnV-glnX*. The genes *glnU* and *glnW* encodes glutamine tRNA having (c)mnm^5^s^2^U34 as wobble nucleoside whereas *glnV* and *glnX* encodes glutamine tRNA having C34 as wobble nucleoside.

b) Plasmid pUST314 carry a part of the *metT* operon due to a spontaneous recombination between the *metT* and *metU* genes. It therefore lacks *leuW-glnU-glnW* genes and contains only the *metT/U-glnV-glnX*. The *glnV* and *glnX* genes encodes gln-tRNA having C34 as wobble nucleoside. Thus this plasmid lacks genes for the gln-tRNA havning (c)mnm^5^s^2^U as wobble nucleoside.

c) Plasmid p815 contains the *valU*-operon which contains the *valU-valX-valY* and *lysV* tRNA genes. The *lysV* gene encodes lys-tRNA having mnm^5^s^2^U34 as wobble nucleoside.

On minimal medium we also observed a severe growth reduction by overexpression of ${\text{tRNA}}_{\text{cmnm}5\text{s}2\text{UUG}}^{\text{Gln}}$ in all mutants lacking the s^2^-group of (c)mnm^5^s^2^U34 but not in the wild type control. A similar growth reduction was also observed with plasmid pUST314, which does not encode ${\text{tRNA}}_{\text{cmnm}5\text{s}2\text{UUG}}^{\text{Gln}}$ but overexpressed ${\text{tRNA}}_{\text{ac}4\text{CAU}}^{\text{Met}}$ and ${\text{tRNA}}_{\text{CUG}}^{\text{Gln}}$. Therefore, we cannot attribute this growth reduction to the overexpression of ${\text{tRNA}}_{\text{cmnm}5\text{s}2\text{UUG}}^{\text{Gln}}$ but to the s^2^-deficiency of (c)mnm^5^s^2^U34 in the cell, since a similar growth reduction was not observed in the wild type strain (Table 3). Apparently, overexpression of the tRNAs from these two plasmids (+/- expression of ${\text{tRNA}}_{\text{cmnm}5\text{s}2\text{UUG}}^{\text{Gln}}$) induces a severe growth reduction. Since this was observed on minimal medium, an unbalanced tRNA population due to the overexpression of the tRNAs encoded by these plasmids might induce in s^2^-deficient strains aberrations of translation of several genes involved in the synthesis of metabolites resulting in multiple auxotrophy.

#### Overexpression of ${\mathrm{tRNA}}_{\mathrm{cmnm}5s2\mathrm{UUG}}^{\mathrm{Gln}}$ lacking the cmnm^5^-group and possessing only the s^2^-group of (c)mnm^5^s^2^U34

Plasmid pUST313 was introduced to the *mnmE13* and *mnmG1* mutants, which both lack the cmnm^5^-group of cmnm^5^s^2^U34 in their ${\text{tRNA}}_{\text{cmnm}5\text{s}2\text{UUG}}^{\text{Gln}}$, and have instead s^2^U34 as wobble nucleoside. This caused also a growth reduction on rich medium although not to the same extent as when this plasmid was introduced into cells lacking the s^2^-group of (c)mnm^5^s^2^U34 (Table 3). Since a similar result was obtained in two different mutants (*mnmE13* or *mnmG1*) inducing a similar mnm^5^- group deficiency, the growth reduction is likely due to the hypomodified ${\text{tRNA}}_{\text{cmnm}5\text{s}2\text{UUG}}^{\text{Gln}}$ and not to some other aberrations in these cells. There was no or very little growth reduction caused by plasmid pUST314, which lacks the genes encoding ${\text{tRNA}}_{\text{cmnm}5\text{s}2\text{UUG}}^{\text{Gln}}$. Thus, the decreased growth observed is caused by overexpression of hypomodified ${\text{tRNA}}_{\text{cmnm}5\text{s}2\text{UUG}}^{\text{Gln}}$. Note, that the observed growth reduction on minimal medium was larger than on rich medium and it was dependent on overexpression of ${\text{tRNA}}_{\text{cmnm}5\text{s}2\text{UUG}}^{\text{Gln}}$. Thus, overexpression of ${\text{tRNA}}_{\text{cmnm}5\text{s}2\text{UUG}}^{\text{Gln}}$ lacking the cmnm^5^-group causes a substantial growth reduction on rich medium and even a more severe growth reduction on minimal medium. We suggest that ${\text{tRNA}}_{\text{cmnm}5\text{s}2\text{UUG}}^{\text{Gln}}$ lacking the cmnm^5^-group induces missense errors. Interestingly, ${\text{tRNA}}_{\text{mnm}5\text{s}2\text{UUU}}^{\text{Glu}}$ lacking the mnm^5^-group also increases misreading of GGA (Gly), GAU (Asp) and GAC (Asp) codons [8].

#### Overexpression of ${\mathrm{tRNA}}_{\mathrm{mnm}5s2\mathrm{UUU}}^{\mathrm{Lys}}$ having only the mnm^5^- or the s^2^-group of mnm^5^s^2^U34

Plasmid p815 contains the *valU* operon, which consists of the genes *valU*, *valX*, *valY* and *lysV*, the latter of which is the structural gene for ${\text{tRNA}}_{\text{mnm}5\text{s}2\text{UUU}}^{\text{Lys}}$. Mutations *mnmA3* or *mnmE13* influence only the structure of the ${\text{tRNA}}_{\text{mnm}5\text{s}2\text{UUU}}^{\text{Lys}}$. Overexpression of these tRNAs, including ${\text{tRNA}}_{\text{mnm}5\text{s}2\text{UUU}}^{\text{Lys}}$, resulted in a severe growth reduction of strains lacking the s^2^-group of mnm^5^s^2^U34, suggesting that overexpressing thiol-deficient ${\text{tRNA}}_{\text{mnm}5\text{s}2\text{UUU}}^{\text{Lys}}$ may induce translational errors, such as missense errors (Table 3). However, Hagervall et al [7] showed that s^2^-deficiency of mnm^5^s^2^U34 in ${\text{tRNA}}_{\text{mnm}5\text{s}2\text{UUU}}^{\text{Lys}}$ results in less missense errors in reading the AAU/C (Asn) codons. If our results reflect increased missense errors, they may be due to other such errors than those monitored by Hagervall et al [7]. If so, the missense errors induced by s^2^-deficiency of mnm^5^s^2^U34 in ${\text{tRNA}}_{\text{mnm}5\text{s}2\text{UUU}}^{\text{Lys}}$ may be codon specific, which has been observed earlier [8]. However, such a growth reduction was not observed when the overexpressed ${\text{tRNA}}_{\text{mnm}5\text{s}2\text{UUU}}^{\text{Lys}}$ was mnm^5^-deficient, suggesting that this deficiency did not induce any increased translational errors such as missense error. This result is indeed consistent with earlier reports showing that mnm^5^-deficient ${\text{tRNA}}_{\text{mnm}5\text{s}2\text{UUU}}^{\text{Lys}}$ results in less missense errors in decoding AGA/G (Arg) or AAU/C (Asn) codons [7, 8]. These results strengthen our suggestion that the growth reduction observed by overexpressing hypomodified tRNAs may be correlated to increased missense errors.

The combined results above show that overexpressed s^2^-deficent ${\text{tRNA}}_{\text{cmnm}5\text{s}2\text{UUG}}^{\text{Gln}}$ and ${\text{tRNA}}_{\text{mnm}5\text{s}2\text{UUU}}^{\text{Lys}}$ imposed a considerable growth reduction suggesting that such hypomodified tRNAs increase missense error. Overexpression of ${\text{tRNA}}_{\text{cmnm}5\text{s}2\text{UUG}}^{\text{Gln}}$ lacking the cmnm^5^-group reduced growth although much less than s^2^-deficiency, whereas lack of the mnm^5^ group of overexpressed ${\text{tRNA}}_{\text{mnm}5\text{s}2\text{UUU}}^{\text{Lys}}$ did not.

#### Presence of the wobble (c)mnm^5^s^2^U34 in tRNAs specific for Gln, Lys, and Glu is essential for viability of *S*. *enterica*

To construct a double mutant *mnmA*, *mnmE* we wanted to combine strains harboring both the selectable *mnmA16<>cat* (Cm^R^) and *mnmE17<>Km* (Km^R^) mutations and such a double mutant should have tRNAs specific for Gln, Lys, and Glu with an unmodified wobble uridine (U34). We grew the *mnmA16<>cat* mutant in rich medium at 37°C and mixed the cells with the transducing phage P22 grown on the *mnmE17<>Km*. We then spread the mixture on rich agar plates containing kanamycin to select for the *mnmE17<>Km* mutation and thereby creating the wanted double mutant *mnmA16<>cat*, *mnmE17<>Km*. The Km^R^ transductants were selected at several temperatures to monitor if a double mutant might be viable at another temperature than 37°C. The donor strain GT8176 (STM3453-2550::Tn*10*dTc) also contains a selectable (tetracycline resistant, Tc^R^) mutation known not to induce any growth defect and this mutation was used to monitor the efficiency of transduction. As recipient we also used strain GT8173 (p*mnmA*^*+*^/ *mnmA16<>cat*), which contains the same *mnmA16<>cat* mutation on the chromosome and the complementing plasmid p*mnmA*^*+*^ containing the wild type allele of the *mnmA* gene. Such strain should behave as the wild type unless the mutation *mnmA16<>cat* introduces some unknown phenotype, which is not related to the mutation in *mnmA* gene and therefore not complemented by the p*mnmA*^*+*^ plasmid.

Table 4 shows that no double mutant *mnmA16<>cat*, *mnmE17<>Km* was obtained at any temperature clearly demonstrating that such a double mutant is not viable. This result cannot be due to an inefficient transduction, since the transfer of the STM3453-2550::Tn*10*dTc marker was similar to the transfer frequency of the *mnmE17<>Km* mutation to the wild type (Compare the number of Tc^R^ colonies to the number of Km^R^ colonies in the wild type). Moreover, the transduction frequency of *mnmE17<>Km* was similar when strain GT8177 (p*mnmA*^*+*^/ *mnmA16<>cat*) was used as recipient. Note also, that we waited several days in order to allow a very slow growing double mutant to appear but still no such mutant was recovered. Moreover, we did not obtain any external suppressor mutant with mutation counteracting the lack of modification of U34. We conclude that cells having an unmodified wobble uridine of tRNAs specific for Gln, Lys and Glu are not viable on rich medium at temperatures between room temperature (about 20°C) and 42°C.

**Table 4:** The double mutant *mnmA*, *mnmE* is nonviable on rich medium at 15 to 37°C.

Temp (°C)	Donor (Relevant genotype)	Recipient (Relevant genotype)	Tc^R^ No. of colonies (days)	Km^R^ No. of colonies (days)
37	**GT8176**(*mnmE17<>Km*, STM3453-2550::Tn*10*dTet)	**GT7321**(*mnmA*^*+*^*mnmE*^+^)	976 (3)	691 (3)
37	“	**GT8177** (*pmnmA*^*+*^/ *mnmA16<>cat*)	463 (3)	393 (3)
37	”	**GT8173** (*mnmA16<>cat*)	915 (3)	**0** (7); 2^a^
30	”	**GT7321**(*mnmA*^*+*^*mnmE*^*+*^*)*	584 (3)	308 (5)
30	”	**GT8173** (*mnmA16<>cat*)	628 (3)	**0** (29)
RT	”	**GT7321**(*mnmA*^*+*^*mnmE*^+^)	521 (4)	191 (14)
RT	”	**GT8173** (*mnmA16<>cat*)	482 (6)	**0** (35)
15	”	**GT7321**(*mnmA*^*+*^*mnmE*^*+*^)	155 (10)	28 (10)
15	”	**GT8177** (p*mnmA*^*+*^/ *mnmA16<>cat*)	224 (10)	80 (10)
15	”	**GT8173** (*mnmA16<>cat*)	**0** (44)	**0** (44)

Recipient cells were grown in rich medium at 37°C. Following transduction using phage P22 grown on indicated donor strains, selection was made on rich plates containing either Km (50 ug/ml) or Tc (20ug/ml). Selection for Tc^R^ colonies shows the efficiency of transduction. The plates were incubated at indicated temperatures for indicated days (in parenthesis) when the number of colonies was scored. Plasmid p*mnmA*^+^ is the pNTR-SD *mnmA*^*+*^ plasmid harbouring the wild type allele of the *mnmA*^*+*^ gene from *E*. *coli* [45].

a) Two small colonies were observed after seven days but they were not analysed further due to their instability and poor growth.

The inability to obtain any transductants at 15°C when selecting Tc^R^ transductants (STM3453-2550::Tn*10*dTc) shows that the recipient *mnmA16<>cat* mutant is not able to grow at such low temperature consistent with the results shown in Table 2.

## Discussion

We [5] and others [32]) have shown that the major function of mcm^5^s^2^U34 in yeast tRNAs is to improve the efficiency of the cognate anticodon-codon interaction rather than preventing missense errors, since overexpression of hypomodified tRNAs specific for Gln, Lys or Glu counteracts the phenotypes induced by deficiency of either s^2^, mcm^5^- or both modifications. However, overexpression of bacterial ${\text{tRNA}}_{\text{cmnm}5\text{s}2\text{UUG}}^{\text{Gln}}$ did not counteract the slow growth caused by s^2^- or cmnm^5^- deficiency but rather exaggerated the reduced growth of these mutants (Table 3). This growth reduction is is caused by the s^2^- or cmnm^5^- deficiency and not to some other cellular aberrations, since the same result was obtained by three independent mutants (*mnmA3*, *tusB27* and *tusE30*) defective in the thiolation and two independent mutants (*mnmE13* and *mnmG1*) defective in the synthesis of the side chain (Table 3). Moreover, this severe growth reduction by overexpression of ${\text{tRNA}}_{\text{cmnm}5\text{s}2\text{UUG}}^{\text{Gln}}$ is due to the surplus of this particular tRNA, since a spontaneous faster growing mutant derivative was obtained that have a plasmid derivative lacking genes *glnU/W*, which both encode this tRNA (Fig 5). These results suggest that the major impact of hypomodified ${\text{tRNA}}_{\text{cmnm}5\text{s}2\text{UUG}}^{\text{Gln}}$ caused increased translational errors, such as missense errors, rather than reduced efficiency of reading the cognate codons as was suggested for yeast tRNA lacking mcm^5^s^2^U34 [5]. If the function of (c)mnm^5^s^2^U34 were to improve the efficiency of cognate codon reading, increased concentration of hypomodified tRNA should rather counteract the reduced growth of the *mnmA3* and *mnmE13* mutants than to exaggerate it. Thus, although the xm^5^s^2^U34 wobble modifications are universally conserved, their functional impacts may be different, at least in yeast and in *S*.*enterica*. A difference in translation accuracy between yeast and *E*. *coli* has been noted earlier, since missense errors are about 10-fold lower in yeast compared to *E*. *coli*. Yeast might have evolved mechanism(s) not present in bacteria to reduce missense errors [33].

An unmodified wobble uridine is rarely present in any cytosolic tRNAs [2]. In organelles, like mitochondria, unmodified uridines are present in tRNAs reading family boxes but not in tRNAs specific for Gln, Lys, and Glu, which always have an xm^5^s^2^U34 derivative. It was thought that the presence of this modification in the wobble position of Gln, Lys and Glu tRNAs would prevent missense errors [4]. The phenotypes of the *mnmA3* and *mnmE17* mutants show that these modifications are pivotal for cellular growth (Table 2) and, indeed, a double mutant *mnmA16<>cat*, *mnmE17<>Km*, which has an unmodified U34 as wobble nucleoside, did not grow at several different temperatures (Table 4). These results are consistent with an earlier report that showed that a similar double mutant of *E*. *coli* is not able to grow [34]. Clearly, the functional impact of (c)mnm^5^s^2^U34 is of the outmost importance and explain why such a modification is always present in these kind of tRNAs and predicted to be present in a minimal translation apparatus [13]. Based on the results presented in this paper we suggest that the major reason for inability to support growth with an unmodified uridine in the wobble position of tRNAs specific for Gln, Lys, and Glu is increased missense errors.

It has been suggested that the s^2^- and xm^5^- groups of xm^5^s^2^U derivatives present in the wobble position would prevent missense errors [4, 35]. This kind of modification is present in split codon boxes (Fig 2) and thus should prevent decoding the near-cognate pyrimidine ending codons in such boxes and thereby missense errors. Although the three tRNAs specific for Gln, Lys and Glu all have the xm^5^s^2^U34 wobble modification they still are part of distinct structural context. The ${\text{tRNA}}_{\text{mnm}5\text{s}2\text{UUU}}^{\text{Lys}}$ has a very flexible anticodon loop due to the presence of several U (a poor stacker) including U36, whereas Gln has G36 and Glu has C36. Moreover, ${\text{tRNA}}_{\text{mnm}5\text{s}2\text{UUU}}^{\text{Lys}}$ has the large hydrophobic cyclic N6-threonylcarbamoyladenosice (ct^6^A) in position 37 and both ${\text{tRNA}}_{\text{cmnm}5\text{s}2\text{UUG}}^{\text{Gln}}$ and ${\text{tRNA}}_{\text{mnm}5\text{s}2\text{UUU}}^{\text{Glu}}$ have the much smaller methylated derivative m^2^A. The ct^6^A improves the stacking interaction with the base above (A38) and below (A36) of the anticodon loop but it also makes a cross strand stack to the first base (A) of the codon and thereby stabilizes the weak A-U36 base pairing in the first position [36, 37]. This strong functional impact on the stability of U-rich anticodon loops may explain why this modification is essential for viability [38]. The functional impact of m^2^A, which is present in ${\text{tRNA}}_{\text{cmnm}5\text{s}2\text{UUG}}^{\text{Gln}}$ and ${\text{tRNA}}_{\text{mnm}5\text{s}2\text{UUU}}^{\text{Lys}}$, is likely to be different and should have a lesser functional impact than ct^6^A37 and accordingly it is not essential for viability [39]. Thus, the structural context for these xm^5^s^2^U wobble modifications is different in these three tRNAs not only because differences in the primary sequence of the anticodon loop but also because of differences in the modification pattern and in particular the nature of the modifications at position 37 (ct^6^A in ${\text{tRNA}}_{\text{mnm}5\text{s}2\text{UUU}}^{\text{Lys}}$ and m^2^A in ${\text{tRNA}}_{\text{cmnm}5\text{s}2\text{UUG}}^{\text{Gln}}$ and ${\text{tRNA}}_{\text{mnm}5\text{s}2\text{UUU}}^{\text{Glu}}$, see Fig 2). These features may influence weather the functional impact by (c)mnm^5^s^2^U34 is primarily to prevent missense errors or to improve the efficiency of cognate reading. The frequency of missense errors also depends on the efficiency of the competing near-cognate tRNAs [40]. Such a consideration may explain that missense errors for hypomodified ${\text{tRNA}}_{\text{mnm}5\text{s}2\text{UUU}}^{\text{Lys}}$ are decreased [7, 8] (Table 3), whereas they are increased for ${\text{tRNA}}_{\text{cmnm}5\text{s}2\text{UUG}}^{\text{Gln}}$ (Table 3) and ${\text{tRNA}}_{\text{mnm}5\text{s}2\text{UUU}}^{\text{Glu}}$ [8]. Note also, that when a specific missense error is monitored it is codon dependent but if growth reduction caused by overexpression of a specific hypomodified tRNA monitors missense errors it will be the total outcome of many possible such errors of several near- and/or non-cognate codons. Therefore, one would not expect identical results using two such different methods to monitor translational errors. Still, in the case of the functional impact of the mnm^5^-side chain on ${\text{tRNA}}_{\text{mnm}5\text{s}2\text{UUU}}^{\text{Lys}}$ the two methods gave consistent results (Table 3 and [7, 8]).

We have suggested that the reduced growth linked to the overexpression of hypomodified ${\text{tRNA}}_{\text{cmnm}5\text{s}2\text{UUG}}^{\text{Gln}}$ or ${\text{tRNA}}_{\text{mnm}5\text{s}2\text{UUU}}^{\text{Lys}}$ is mainly due to increased missense errors, although we cannot exclude other translational errors, such as errors in the reading frame maintenance, misfolding of nascent proteins, or ribosomal drop off. The latter probably does not explain the observed growth reduction, since overexpression of tRNA would counteract such errors. Deficiency of either s^2^- or (c)mnm^5^-group of (c)mnm^5^s^2^U34 induces frameshifts by the peptidyl-slippage mechanism (reviewed in [1]) and according to this model overexpression of the tRNA reading the next codon downstream of the frameshifting site decreases frameshifting. Still, Pande et al [41] noticed that overexpression of a few specific yeast tRNAs increases frameshifts probably by an out-of-frame binding, which pulls the ribosome into the shifted frame. The increase in frameshifting was modest and no major effect on growth was reported. Overexpression of hypomodified tRNA specific for Gln, Lys and Glu in yeast counteracts the phenotypes induced by modification deficiency and does not induce any growth defect [5, 32]. Moreover, as in bacteria overexpression of fully modified yeast tRNA does not cause any reduction in growth. Note also that one of the most efficient frameshift suppressors (*sufA6*) does not in any major way influence cellular growth (unpublished observation). Taken together, increased frameshifting as an explanation to the severe growth reduction observed upon overexpression of hypomodified tRNAs is not likely. On the other hand the growth reduction may cause an aberrant folding of several proteins. The nascent polypeptide is folded on the ribosome and a change of the polypeptide synthesis rate may influence the folding of proteins [42]. The speed with which the mRNA is decoded is correlated to the concentration of cognate tRNAs [43, 44]. Overexpression of an unmodified tRNA may change the rate of polypeptide synthesis and thereby be influencing the folding of the nascent peptides resulting in an accumulation of misfolded proteins. Such aberrant proteins may in turn induce a growth defect. Still, overexpression of fully modified tRNA did not influence the growth (Table 3), a condition which also should change the rate of polypeptide synthesis due to increased concentration of some tRNAs. Therefore, if the observed reduction of growth is caused by misfolding of proteins, it would be specific for modification deficiency. Overexpression of normal or hypomodified yeast tRNAs specific for Gln, Lys and Glu does not reduce cellular growth but rather counteracts the modification deficient induced phenotype(s). Thus in yeast overexpression of hypomodified tRNAs does not induce frameshifts or aberrant folding of proteins to such a degree that it influences growth, although both kinds of translational errors may well be operating also in this organism [5, 32]. From these considerations we favor missense errors causing the observed growth reduction, since theoretical considerations have suggested that the function of the xm^5^s^2^U34 is to prevent such errors. Moreover, such errors have been observed experimentally [8] although in some experiments reduced missense errors by hypomodified tRNA was noticed [7, 8]. These experiments monitored effects on specific codons why it is not excluded that misreading of other codons may be operating. Overexpression of ${\text{tRNA}}_{\text{mnm}5\text{s}2\text{UUU}}^{\text{Lys}}$ lacking the mnm^5^-group did not reduce growth suggesting no increase missense errors consistent with results obtained earlier [7, 8], Thus, monitoring growth may well be relevant to estimate missense errors although not as specific as a direct measure of missense errors. Even if we cannot rule out other translational errors, we still found it likely that the growth reduction observed is caused mainly by increased missense errors. This suggestion is in line with the fact that the xm^5^s^2^U34 wobble modification have been shown theoretically and experimentally to influence the accuracy of translation besides its effect on the efficiency of cognate codon reading. Therefore, the primary function of the xm^5^s^2^U34 modifications may be different in yeast and in bacteria.

## Supporting information

S1 Table Strains and plasmids. (DOCX)
