# Supplementary material for: An unmodified wobble uridine in tRNAs specific for Glutamine, Lysine, and Glutamic acid from Salmonella enterica Serovar Typhimurium results in nonviability—Due to increased missense errors?
Source: PLoS One. 2017 Apr 21;12(4):e0175092. doi: 10.1371/journal.pone.0175092 (PMC5400242; doi:10.1371/journal.pone.0175092)
Supplement: S1 Table — (DOCX) [file pone.0175092.s001.docx]

| Strains |  |  |
| --- | --- | --- |
| GT7321 | *hisD10122* (ccccaa), *zdd-2532*::cat, *hisO1242* | Laboratory collection |
| GT7432 | *tusE30*, STM1091-2549::Tn*10*dTet, *hisD10122*(ccccaa), *zdd-2532*::cat, *hisO1242* | ” |
| GT7436 | *mnmE13* (deletion aa247-271), STM3848/3849-2542::Tn*10*dTet, *hisD10122*, *zdd-2532*::cat, *hisO1242* | “ |
| GT7440 | *mnmA3*, STM1221-2545::Tn*10*dTet, *hisD10122*(ccccaa), *zdd-2532*::cat, *hisO1242* | “ |
| GT7453 | *tusB27*, STM3453-2550::Tn*10*dTet, *hisD10122*(ccccaa), *zdd-2532*::cat, *hisO1242* | “ |
| GT7478 | *gidA1*(58bp del from aa402), STM3848/3849-2542::Tn*10*dTet, *hisD10122*(ccccaa), *zdd-2532*::cat, *hisO1242* | “ |
| GT8173 | *mnmA16*<>cat, *hisD10122*(ccccaa), *zdd-2532*::cat, *hisO1242* | This work |
| GT8176 | *mnmE17*<>kan, STM3453-2550::Tn*10*dTet, *hisD10122*(ccccaa), *zdd-2532*::cat, *hisO1242* | “ |
| GT8177 | pmnmA / *mnmA16*<>cat, *hisD10122*(ccccaa), *zdd-2532*::cat, *hisO1242* | “ |
| GT8041 | pUST312 / *hisD10122*(ccccaa), *zdd-2532*::cat, *hisO1242* | “ |
| GT8042 | pUST312 / *rpsI2*, STM3333- 2543::Tn*10*dTet, *hisD10122*(ccccaa), *zdd-2532*::cat, *hisO1242* | “ |
| GT8046 | pUST312 / *mnmA3*, STM1221-2545::Tn*10*dTet, *hisD10122*(ccccaa), *zdd-2532*::cat, *hisO1242* | “ |
| GT8047 | pUST312 / *mnmE13*, STM3848/3849-2542::Tn*10*dTet, *hisD10122*(ccccaa), *zdd-2532*::cat, *hisO1242* | “ |
| GT8048 | pUST312 / *tusB27*, STM3453-2550::Tn*10*dTet, *hisD10122*(ccccaa), *zdd-2532*::cat, *hisO1242* | “ |
| GT8049 | pUST312 / *tusE30*, STM1091-2549::Tn*10*dTet, *hisD10122*(ccccaa), *zdd-2532*::cat, *hisO1242* | “ |
| GT8050 | pUST312 / gidA1, STM3848/49-2542::Tn*10*dTet, *hisD10122*(ccccaa), *zdd-2532*::cat, *hisO1242* | “ |
| GT8069 | pUST313 / *hisD10122*(ccccaa), *zdd-2532*::cat, *hisO1242* | “ |
| GT8070 | pUST313 / *rpsI2*, STM3333-2543::Tn*10*dTet, *hisD10122*(ccccaa), *zdd-2532*::cat, *hisO1242* | “ |
| GT8074 | pUST313 / *mnmA3*, STM1221-2545::Tn*10*dTet, *hisD10122*(ccccaa), *zdd-2532*::cat, *hisO1242* | “ |
| GT8075 | pUST313 / *mnmE13*, STM3848/49-2542 ::Tn*10*dTet, *hisD10122*(ccccaa), *zdd-2532*::cat, *hisO1242* | “ |
| GT8076 | pUST313 / *tusB27*, STM3453-2550::Tn*10*dTet, *hisD10122*(ccccaa), *zdd-2532*::cat, *hisO1242* | “ |
| GT8077 | pUST313/ *tusE30*, STM1091-2549::Tn*10*dTet, *hisD10122*(ccccaa), *zdd-2532*::cat, *hisO1242* | “ |
| GT8078 | pUST313 / *gidA1*, STM3848/49-2542::Tn*10*dTet, *hisD10122*(ccccaa), *zdd-2532*::cat, *hisO1242* | “ |
| GT8156 | pUST314 / *mnmA3*, STM2220-2546::Tn*10*dTet, *hisD10122*(ccccaa), *zdd-2532*::cat, *hisO1242* | “ |
| GT8157 | pUST314 / *mnmE13*, STM3848/49-2542 ::Tn*10*dTet, *hisD10122*(ccccaa), *zdd-2532*::cat, *hisO1242* | “ |
| GT8158 | pUST314 / *hisD10122*(ccccaa), *zdd-2532*::cat, *hisO1242* | “ |
| GT8296 | pUST314/ *tusB27*, STM3453-2550::Tn*10*dTet, *hisD10122*(ccccaa), *zdd-2532*::cat, *hisO1242* | “ |
| GT8297 | p815/ *tusB27*, STM3453-2550::Tn*10*dTet, *hisD10122*(ccccaa), *zdd-2532*::cat, *hisO1242* | “ |
| GT8295 | p815/*hisD10122* (ccccaa), *zdd-2532*::cat, *hisO1242*, *mnmA3*, STM1221::Tn*10*dTet | “ |
| GT8298 | p815/ *tusE30*, STM1091-2549::Tn*10*dTet, *hisD10122*(ccccaa), *zdd-2532*::cat, *hisO1242* | “ |
| GT8299 | p815/ *mnmE13* (deletion aa247-271), STM3848/3849-2542::Tn*10*dTet,*hisD10122*, *zdd-2532*::cat, *hisO1242* | “ |
| GT8300 | p815/ *gidA1*(58bp del from aa402-), STM3848/3849-2542::Tn*10*dTet, *hisD10122*(ccccaa), *zdd-2532*::cat, *hisO1242* | “ |
| Plasmids |  | “ |
| pmnmA | pNTR-SD-*mnmA* under IPTG inducible promoter, Amp^R^ | 1 |
| p815 | *E.coli* *valU* operon in pLG339, Km^R^ | 2 |
| pUST312 | pLG339 with 10 bp inserted in the BamHI site creating a SalI site, Km^R^ | This work |
| pUST136 | pLG339 containing a 5.5 kB Sau3A fragment with i.a. *metT*,*leuW*,*glnUW,metU,glnVX* genes, Km^R^ | 3 |
| pUST313 | pUST136 cut with BamHI and SalI and religated to give a fragment with the tRNA genes from pUST136, Km^R^ | This work |
| pUST314 | Revertant of pUST313 with only *metT,metU,glnVX* genes, Km^R^ | This work |

|  |  |  |  |  |  |  |
| --- | --- | --- | --- | --- | --- | --- |

**References.**

1. Saka, K, Tadenuma, M, Nakade, S, Tanaka, N, Sugawara, H, Nishikawa, K, Ichiyoshi, N, Kitagawa, M, Mori, H, Ogasawara, N, Nishimura, A. (2005) A complete set of Escherichia coli open reading frames in mobile plasmids facilitating genetic studies. DNA Res. 12, 63-68.
2. O'Connor, M. Insertions in the anticodon loop of tRNAGln1(*sufG*) and tRNALys promote quadruplet decoding of CAAA (2002). Nucl Acids Res 30, 1985-1990.

1. Esberg, B, Leung, HC, Tsui, HC, Björk, GR, Winkler, ME (1999) Identification of the *miaB* gene, involved in methylthiolation of isopentenylated A37 derivatives in the tRNA of *Salmonella typhimurium* and *Escherichia coli*. J. Bacteriol. 181, 7256-7265.
